# Can an FDA-Approved Alzheimer’s Drug Be Repurposed for Alleviating Neuronal Symptoms of Zika Virus?

**DOI:** 10.1128/mBio.00916-17

**Published:** 2017-06-27

**Authors:** Devika Sirohi, Richard J. Kuhn

**Affiliations:** Department of Biological Sciences, and Purdue Institute of Inflammation, Immunology and Infectious Disease, Purdue University, West Lafayette, Indiana, USA

**Keywords:** Zika virus, antiviral agents, neurodegeneration, NMDAR, memantine

## Abstract

Zika virus caught the world by surprise by its rapid spread and frightening disease outcomes. This major epidemic motivated many scientists to focus their attention on controlling this emerging pathogen. As many as 45 vaccine candidates are being developed, but progress in the antiviral arena has been slower. In a recent article (mBio 8:e00350-17, 2017, https://doi.org/10.1128/mBio.00350-17), Costa and colleagues showed that an FDA-approved drug used to treat Alzheimer’s disease may moderate Zika virus-induced neuronal damage. This work is based on the premise that overstimulation of *N*-methyl-d-aspartate receptors (NMDARs) may drive neurodegeneration and that this may be responsible for neuronal cell death associated with Zika virus infection of the central nervous system (CNS). Thus, blockage of the NMDAR channel activity with FDA-approved memantine or other antagonists may reduce neurological complications associated with Zika virus infection. Repurposing a preapproved drug and targeting the host represent intriguing strategies and yet require more analysis prior to moving into clinical trials.

## COMMENTARY

Zika virus, an exemplar reemerging virus, poses an enormous global threat that is aggravated by its unique sexual and mosquito vector transmission. Zika virus was discovered in 1947 but remained quiescent for more than half a century, with only a few reported cases of mild human infections. The virus resurfaced recently and initiated severe epidemics in South America and the Pacific islands, where an increase in the incidence of neonatal microcephaly and of autoimmune Guillain-Barré syndrome was observed. Much of what we know about the virus has been gathered in the wake of the epidemic. Data from clinical studies, animal models, and cell culture experiments have proven beyond reasonable doubt that Zika virus is neurotropic and causes a wide spectrum of neurodevelopmental defects in the human fetus, now grouped under the umbrella of congenital Zika syndrome. These include, but are not limited to, microcephaly, ventriculomegaly, hydrocephalus, intracranial calcifications, arthrogryposis, fetal brain disruption sequence, intrauterine fetal growth restriction, and a range of visual, auditory, motor, and cognitive deficits (1). Numerous candidate vaccines against Zika virus are being evaluated in clinical trials, but therapeutic options appear further out of sight. Efforts are being made to repurpose FDA-approved drugs to fast-track the drug development pipeline for control of Zika virus. One such attempt is described in a recent article by Costa and colleagues (2). The group tested the protective efficacy against Zika virus infection of memantine, which is an FDA-approved *N*-methyl-d-aspartate receptor (NMDAR) antagonist for the treatment of moderate-to-severe Alzheimer’s disease. The underlying hypothesis for the study was that Zika virus infection, similarly to Alzheimer’s disease and other neurodegenerative diseases, causes an overstimulation of NMDAR, leading to glutamate excitotoxicity and neuronal death. Blocking hyperactivation of NMDAR would therefore reduce rates of Zika virus-induced cell death and help ameliorate neuronal symptoms during infection.

**FIG 1:**
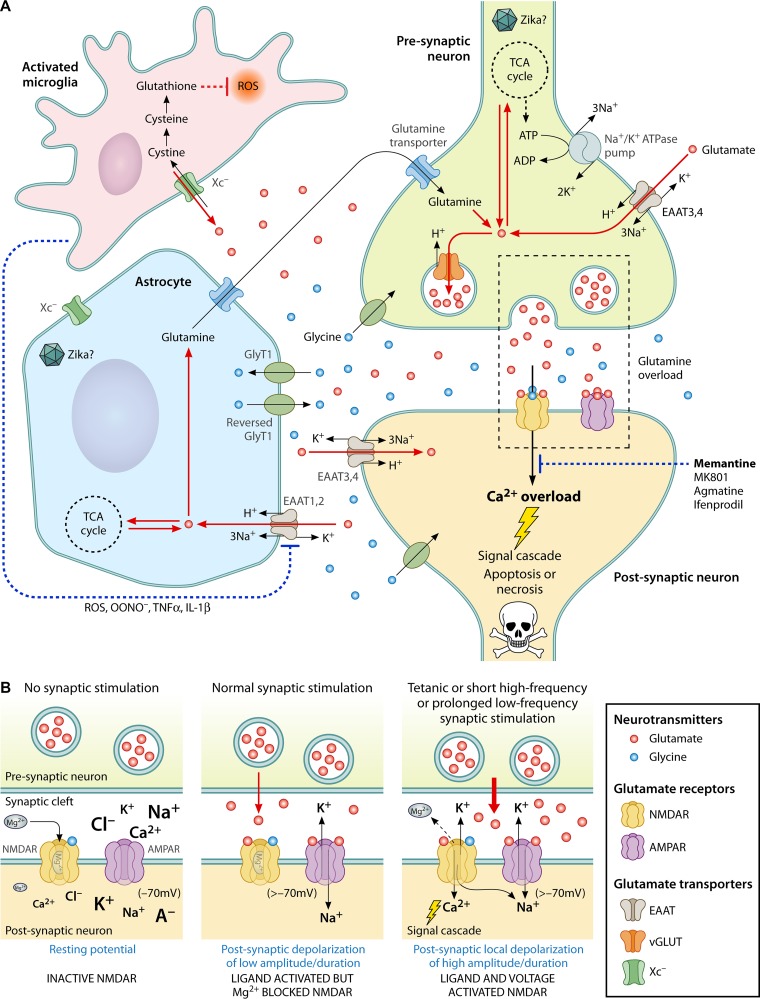
Glutamate neurotransmission, homeostasis, and excitotoxicity in the brain. (A) A glutamate-driven chemical synapse along with a neighboring astrocyte and an activated microglia are shown. Under physiological conditions, a depolarizing impulse on the presynaptic neuron triggers a transient spike in the concentration of glutamate in the synaptic cleft. Glutamate binds to NMDA and AMPA ionotropic glutamate receptor families on the postsynaptic neuron. These receptor channels, when activated, allow the flow of Na^+^-K^+^, leading to an excitatory postsynaptic potential. If the threshold is achieved, an action potential is generated at the axon hillock of the postsynaptic neuron. NMDAR, in addition to Na^+^-K^+^, also allows conductivity of Ca^2+^ ions. Ca^2+^ ions trigger a signaling cascade that helps modulate the strength of the synapse (a magnified view of the inset is provided in panel B). Under neurodegenerative pathological conditions such as those associated with Alzheimer’s disease or, potentially, Zika virus infection, NMDAR may be hyperactivated, leading to Ca^2+^ overload in cells and subsequent death of the postsynaptic neuron through a process termed "glutamate excitotoxicity." Glutamate levels are tightly regulated in the brain. Glutamate does not cross the adult blood-brain barrier and is synthesized in the brain *de novo* from an intermediate of the TCA cycle. It is efficiently and swiftly cleared from the synapses by EAATs, especially EAAT2 (localized on astrocytes). Glutamate is shuttled between astrocytes and neurons by the glutamate-glutamine cycle and is packaged into synaptic vesicles in neurons by vGLUT. Glutamate homeostasis may be perturbed under pathological conditions or during infection by Zika virus by a variety of mechanisms such as (i) oxidative stress, mitochondrial dysfunction, or energy deficiency (via loss of ATP-dependent Na^+^/K^+^ pumps), which can cause chronic or excessive release of glutamate from the presynaptic neurons; (ii) the release of large amounts of glutamate from activated microglia mediated by Xc^−^ antiporters; and (iii) the reduction of glutamate clearance from synaptic clefts due to compromised activity of EAATs on astrocytes. This could occur due to the presence of ROS and inflammatory cytokines produced by activated microglia or due to damage (or possibly due to infection) in astrocytes themselves. The net result is the unprovoked and sustained presence of glutamate in the synaptic cleft and chronic activation of NMDAR, leading to neuronal damage. NMDAR may also be hyperactivated if the ambient glycine levels in the synaptic cleft are perturbed due to malfunctioning of the glycine transporters (GlyT1). NMDAR antagonists such as FDA-approved memantine, MK-801, agmatine, and ifenprodil (specific for NMDAR containing GluN2B subunits) were shown to prevent neuronal damage during Zika virus infection, but the mechanism leading to hyperactivation of NMDAR during Zika virus infection remains unknown. (B) Activation and functioning of ionotropic glutamate receptors NMDAR and AMPAR. Both NMDAR and AMPAR represent ligand gated ion channels that often coexist at synapses and allow flow of cations in accordance with their concentration gradients. The font size of the ions shown is proportional to their relative concentrations to predict the direction of flow of ions across the membrane. NMDAR allows passage of Na^+^, K^+^, and Ca^2+^ ions, while the prevalent GluA2 subunit containing AMPAR is conductive for Na^+^-K^+^ only. NMDAR requires glutamate and glycine for activation, while glutamate is the exclusive ligand for AMPAR. During a normal or weak synaptic event, only the fast AMPAR channels are activated whereas the slow NMDARs, though bound by ligands, remain inactive due to the obstruction imposed by the nonpermeant Mg^2+^ ion. NMDARs are coincidence detectors and require both ligand and voltage for activation. A depolarization of adequate amplitude/duration by neighboring AMPAR channels results in the repulsion of Mg^2+^ ion from the NMDAR channels, which are then opened, allowing the flow of cations, including Ca^2+^. Influx of Ca^2+^ in cells activates a signaling cascade that regulates synaptic strength and plasticity. Abbreviations: NMDA, *N*-methyl-d-aspartate receptor; AMPAR, α-amino-3-hydroxy-5-methyl-4-isoxazolepropionic acid receptor; EAAT, excitatory amino acid transporter; vGLUT, vesicular glutamate transporter; Xc^−^, cystine-glutamate antiporter; GlyT1, glycine transporter; TCA, tricarboxylic acid (cycle); ROS, reactive oxygen species; OONO^−^, peroxynitrite; TNF-α, tumor necrosis factor alpha; IL-1β, interleukin-1β; A^−^, anion; H^+^, hydrogen; Ca^2+^, calcium; Mg^2+^, magnesium; Na^+^, sodium; K^+^, potassium.

Glutamate is the most prominent excitatory neurotransmitter in the brain. It is implicated in neural communication, such as relaying depolarizing electric stimuli across synapses and modulating the strength of synapses crucial for learning and memory. Depolarization of the presynaptic neuronal membrane triggers release of glutamate, which binds and activates the ionotropic group and/or metabotropic group of glutamate receptors on the postsynaptic neuronal membrane (Fig. 1). Ionotropic receptors are ion channels that open upon activation and allow flow of sodium (Na^+^) and potassium (K^+^) ions (with calcium/[Ca^2+^] in some cases) along their electrochemical gradients. This generates a local depolarization or excitatory postsynaptic potential; if the required threshold is achieved, an action potential is generated that transmits across the axon of the postsynaptic neuron. The members of the other group of glutamate receptors, the metabotropic receptors, are not ion channels themselves but are G protein-coupled receptors that indirectly modulate the activity of ion channels through a signaling cascade. There are three ionotropic and eight metabotropic glutamate receptor families with distinct pharmacological, functional, localization, and kinetic profiles. NMDAR belongs to the ionotropic group of glutamate receptors along with the members of the α-amino-3-hydroxy-5-methyl-4-isoxazolepropionic acid receptor (AMPAR) and kainate receptor (KR) families. The families are named according to their differentiating pharmacological agonists. These receptors exist as tetrameric combinations of different subunits: NMDAR (GluN1, GluN2A-D, GluN3A-B), AMPAR (GluA1-4), and KR (GluK1-5). NMDAR is unique and differs from the other ionotropic glutamate receptor families in (i) its requirement for a coagonist, glycine, for activation and (ii) its dependence on voltage for activity—at resting membrane potential, nonpermeant magnesium ion (Mg^2+^) from the extracellular milieu blocks the NMDAR ion channel. Depolarization of adequate strength and duration via activation of fast AMPAR channels is required to expel Mg^2+^ from NMDAR to allow passage of permeant cations; (iii) unlike KR and AMPAR channels containing the GluA2 subtype that have no or minor calcium conductance, NMDAR permits passage of Ca^2+^ in addition to Na^+^ and K^+^ ions. Ca^2+^ influx into the postsynaptic cells triggers a signaling cascade that serves to strengthen (long-term potentiation) or weaken (long-term depression) synapses. Regulating the number and conductance of AMPAR channels and controlling the presynaptic glutamate release via retrograde nitric oxide signaling are two examples of the ways in which the NMDAR-mediated Ca^2+^ signaling governs synaptic plasticity (Fig. 1B).

Glutamate must be present at the right place, at the right time, and in the right amount. Therefore, glutamate homeostasis is tightly controlled (Fig. 1A). The adult blood-brain barrier is largely impervious to glutamate; both astrocytes and neurons in the brain synthesize glutamate *de novo*. Glutamate is produced by transamination of α-ketogluterate, which is an intermediate in the tricarboxylic acid (TCA) cycle. Glutamate is converted to glutamine in astrocytes by glutamine synthetase and released from cells via a glutamine transporter. The reverse happens in neurons (conversion of glutamine to glutamate by glutaminase) followed by packaging of glutamate into synaptic vesicles by vesicular glutamate transporters (vGLUT). The concentrations of glutamate in the cytoplasm of neurons and astrocytes are about 10 and 2 mM, respectively, and can be as high as 100 mM in synaptic vesicles. The concentration of glutamate is very low (<1 μM) in the cerebrospinal fluid and is anticipated to be even lower in the synaptic cleft. Presynaptic depolarization transiently increases the extracellular glutamate levels, but the neurotransmitter is rapidly and efficiently cleared by excitatory amino acid transporters 1 (EAAT1) to EAAT5, with EAAT1 and EAAT2 localized on astrocytes. Astrocytes are essential for maintaining the appropriate levels of extracellular glutamate. Astrocytes can also release glutamate in the extracellular milieu by several methods that are poorly understood, including those associated with Ca^2+^-dependent channels, exocytosis, volume-sensitive organic anion channels (during astrocyte swelling), and reverse activity of EAATs (upon astrocyte damage) (3).

Perturbation in the levels of extracellular glutamate can have pathological consequences. Underactivation of NMDAR can lead to memory and learning dysfunction and diseases such as schizophrenia (4). Overactivation of NMDAR by severe, chronic, and/or sustained glutamate release can lead to Ca^2+^ overload in neurons, which can initiate apoptosis or necrosis in cells (Fig. 1A). This process of neuronal damage or cell death, called glutamate excitotoxicity, has been implicated in the pathophysiology of ischemia, hypoxia, Alzheimer’s disease, Huntington’s disease, and many other neuropsychiatric disorders (5).

Intriguingly, the work reported by Costa and colleagues (2) suggests that glutamate excitotoxicity could also play a role in Zika virus pathogenesis. Treatment with NMDAR antagonists such as the FDA-approved memantine, MK-801, agmatine, and ifenprodil (targeted against GluN2B containing NMDAR), added after attachment of Zika virus to primary neurons, effectively prevented cell death of the neuronal culture *in vitro*, without reducing viral titers. However, administration of memantine 3 days postinfection to alpha/beta interferon (IFN-α/β) receptor-deficient, immunocompromised adult mice (IFN-α/βR^−/−^) yielded mixed results. While the drug treatment was able to suppress the increase of intraocular pressure and the histopathological signs of neurodegeneration and microgliosis observed during Zika virus infection, it failed to prevent loss of body weight or the increase of inflammatory markers and cytokines in the brain. Those authors propose a possible use of memantine in conjunction with antivirals to manage neuronal symptoms during Zika virus infection. Memantine is an uncompetitive inhibitor that is believed to selectively inhibit the activity of a pathological-chronically active NMDAR, while the physiological functioning of NMDAR is left unaffected. Properties such as strong voltage dependence, moderate affinity, and high on/off rates make it superior to both Mg^2+^ and any tight-binding antagonist (such as MK-801) (6). Furthermore, memantine is promising as a drug target for the control of Zika virus as it falls under FDA pregnancy category B (i.e., no evidence of fetal damage in animals is available, but risk has not been rigorously assessed in controlled human trials).

The work by Costa and colleagues is relevant from both the translational and fundamental perspectives. It is currently unknown how Zika virus induces neuronal cell death. If glutamate excitotoxicity is one of the mechanisms, the issue then is that of how Zika virus infection perturbs glutamate homeostasis. Figure 1A highlights some possibilities. Aberrant depolarization and excessive or prolonged glutamate release could be triggered from the presynaptic membranes of Zika virus-infected neurons by oxidative stress, mitochondrial dysfunction, or energy imbalance (via loss of ATP-dependent Na^+^/K^+^ pumps that maintain resting potential) in these cells. Costa et al. reported a dramatic increase in the number of activated tissue-resident macrophages in the brain (called "microglia") during Zika virus infection, a process termed "microgliosis." Activated microglia can also release glutamate in the extracellular milieu via the cystine-glutamate antiporter (Xc^−^). Xc^−^ usually imports one molecule of glutamate into cells for every cystine molecule that is pushed out; the directionality of flow of these molecules may be reversed should excess cystine be required to synthesize the glutathione needed to counteract reactive oxygen species (ROS) produced in activated microglia (7). Furthermore, activated microglia can compromise the functioning of EAATs in astrocytes via ROS and inflammatory cytokines that can further exacerbate the conditions leading to glutamate excitotoxicity. The glutamate levels could also be affected by Zika virus-induced damage to astrocytes. Downregulation of glutamine synthetase and EAAT2 (also called GLT1) in astrocytes has been observed in a neuroinvasive respiratory virus model (8). Investigation of how Zika virus causes an imbalance in glutamate levels needs further experimentation.

The glutamate signal is received by neighboring neurons, many of which may not be infected, and bystander cells can thus die as a result of excitotoxicity during Zika virus infection; measuring the incidence of this bystander cell death would provide valuable information regarding Zika virus pathology. An intriguing observation in the study was the complete rescue of cell death of Zika virus-infected primary neurons *in vitro* by memantine and other NMDAR antagonists administered in various doses. While the possibility of protection of bystander cells mediated by NMDAR antagonists is easier to envision, it is not clear how Zika virus-infected neurons are spared cell death. NMDAR channels are also believed to be present on the presynaptic membranes, but their significance during physiology and pathology is not well understood. The role of pre- and postsynaptic NMDAR in Zika virus pathogenesis requires further investigation.

An important assumption by the authors is that Zika virus infection is a “neurodegenerative” disease, and they have accordingly subscribed to the use of an adult mouse model for their studies. However, Zika virus has been shown to perturb neurodevelopment by primarily infecting neuronal progenitor cells (9). It appears that defects in formation of neurons via loss of progenitor cells represent the primary problem seen during Zika virus infection, and loss of functional neurons/synapses is nominal or secondary. A comparative study of neurotropic the Zika virus and West Nile virus flaviviruses showed that the former favored progenitor cells in the ventricular zone whereas the latter had a bias toward neurons in the intermediate zone and cortical plate of the developing mouse neocortex (10). Though its activity is not well understood, glutamate is believed to play a role in proliferation, migration, and differentiation of neural progenitor cells and also affects adult neurogenesis (11). Furthermore, NMDARs have been shown to be expressed in the cortical progenitor cells of the human fetus (12). Probing the efficacy of NMDAR antagonists in mitigating neuronal disease in an embryonic model system will provide insights of physiological relevance to this work which are crucial before clinical trials are envisaged.
